# Using Ecological Momentary Assessments to Study How Daily Fluctuations in Psychological States Impact Stress, Well-Being, and Health

**DOI:** 10.3390/jcm13010024

**Published:** 2023-12-19

**Authors:** Summer Mengelkoch, Daniel P. Moriarity, Anne Marie Novak, Michael P. Snyder, George M. Slavich, Shahar Lev-Ari

**Affiliations:** 1 Department of Psychiatry and Biobehavioral Sciences, University of California, Los Angeles, CA 90095, USA; 2 Department of Health Promotion, School of Medicine, Tel-Aviv University, Tel-Aviv 6997801, Israel; annemarie@mail.tau.ac.il; 3 Department of Genetics, Stanford University School of Medicine, Stanford, CA 94305, USA; mpsnyder@stanford.edu

**Keywords:** ecological momentary assessment, stress, well-being, health, mood, social safety, energy, present focus, burnout

## Abstract

Despite great interest in how dynamic fluctuations in psychological states such as mood, social safety, energy, present-focused attention, and burnout impact stress, well-being, and health, most studies examining these constructs use retrospective assessments with relatively long time-lags. Here, we discuss how ecological momentary assessments (EMAs) address methodological issues associated with retrospective reports to help reveal dynamic associations between psychological states at small timescales that are often missed in stress and health research. In addition to helping researchers characterize daily and within-day fluctuations and temporal dynamics between different health-relevant processes, EMAs can elucidate mechanisms through which interventions reduce stress and enhance well-being. EMAs can also be used to identify changes that precede critical health events, which can in turn be used to deliver ecological momentary interventions, or just-in-time interventions, to help prevent such events from occurring. To enable this work, we provide examples of scales and single-item questions used in EMA studies, recommend study designs and statistical approaches that capitalize on EMA data, and discuss limitations of EMA methods. In doing so, we aim to demonstrate how, when used carefully, EMA methods are well poised to greatly advance our understanding of how intrapersonal dynamics affect stress levels, well-being, and human health.

## 1. Introduction

Researchers frequently use scales that assess psychological states retrospectively (e.g., “Over the last two weeks, I felt...”) or as a trait (e.g., “In general, I feel...”), even though many psychological states fluctuate both over the course of a day and between days (e.g., [1,2,3,4,5]). In turn, there is strong evidence suggesting that these dynamic fluctuations influence how individuals interpret and respond to ambiguous events in their daily lives [6,7,8]. For example, imagine a scenario in which all your co-workers were invited to a happy hour after work, but you were left off the invitation. If you are in a positive mood, you might perceive this as an oversight; however, if you are in a more negative mood, or in the aftermath of a night of poor sleep, you might perceive this as a purposeful exclusion or a deliberate attack. Because people experience ambiguous events every day—and given that subjective experiences and appraisals of events have been found to be a stronger predictor of psychological and biological outcomes than more objective assessments of life events [9,10]—it is important to better understand how fluctuations in important psychological states influence not just how individuals experience specific events, but also how these changes relate to biological and clinical states over time.

Historically, it has been challenging to capture dynamic variation in psychological states that influence people’s stress, well-being, and long-term health in controlled laboratory experiments. This is due, in part, to the time restrictions and arbitrary settings that reduce external validity inherent to laboratory research and traditional ways of collecting data. In contrast, ecological momentary assessments (EMAs) involve scheduled data collection to be completed outside of the lab in a participant’s natural environment, enabling researchers to assess daily, ecologically valid experiences and fluctuations in psychological states in real time. EMAs can be especially useful when trying to assess associations between psychological states and dynamic outcomes such as stress, well-being, and health, which are difficult to adequately assess using infrequent measurements. In the present article, we discuss how EMAs are especially useful to investigators seeking to understand how dynamic fluctuations in psychological states impact stress, well-being, and health. First, we highlight what is known about how mood, social safety, energy levels, present focus, and burnout influence stress, well-being, and health. Then, we provide examples of how these constructs have been assessed in prior studies. Finally, we discuss the limitations of EMAs, provide recommendations for researchers seeking to employ EMA methods, and discuss future research directions. 

## 2. EMAs

**Figure 1:**
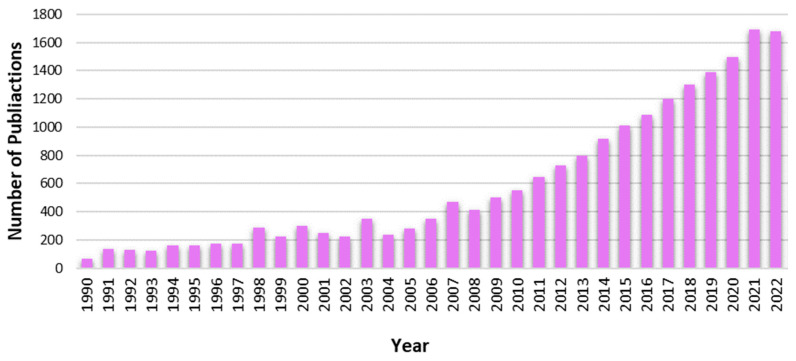
The number of publications containining the term “EMA” has increased over time. Data generated using Web of Science.

EMAs have been used in academic research since the 1990s, although their use has become far more common and effective as recent advances in technology increased accessibility (see Figure 1 for a visualization of the increase in academic research publications that contain the term “EMA” over time). Prior to the adoption of this term and the development of its methodology, similar methods may have been used for momentary assessments in various fields, including for academic research purposes, police investigations, and journalistic reports. Today, EMAs generally take the form of brief surveys that are digitally sent to participants and which are intended to be completed in real time, as individuals are experiencing events in their daily lives. Participants can be assessed at random intervals or in conjunction with key events of interest. Oftentimes, EMAs assess people’s thoughts, feelings, or behaviors, although any variables of interest can be measured. Advantages of EMA survey methods include the reduction in recall bias and erroneous reporting due to forgetfulness, their ecological validity, and the use of repeated assessments over time, the latter of which enables investigations of complex, within-person, temporal interactions between processes such as mood, stressful life events, and health behaviors [11]. Further, the use of EMAs also removes concerns associated with between-person confounders, reduces concerns about time-varying confounders, and can reduce the downward bias on effect sizes induced by overly long assessment lags [12]. 

**Figure 2:**
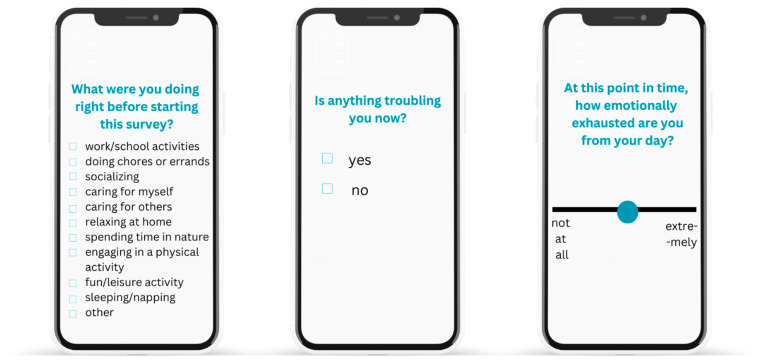
Examples of ecological momentary assessments (EMAs) conducted using mobile devices. Collecting data with multiple quick response types (i.e., multiple input, yes/no, and slider scales) reduces participant burden and allows for the cross-checking of data validity. Additional options, like audio responses and selecting pictures/emoticons as responses, can increase the accessibility of EMA surveys.

Many EMAs are sent to participants as notifications on smartphones and thus assess states in real time. In Figure 2, we show how EMA questions may be presented to participants using a smartphone (based on examples from [13,14,15]). However, other EMA methods are similar to a daily diary method, where participants respond to prompts once per day, reflecting upon their day in general, as opposed to their current state. Finally, EMAs can also be paired with sensing technology using a smartwatch [16], which can enrich these assessments by pairing them with a participant’s physiological state (e.g., heart rate, blood pressure, galvanic skin responses), objective behaviors (e.g., activity levels, social interactions, sleep), and location. 

## 3. Psychological States

Although many psychological states and their daily fluctuations impact stress and well-being, some states have been consistently and strongly related to stress levels, well-being, and health, and are thus particularly important to measure at a high frequency in participants’ daily lives. First, we discuss well-being and stress. Next, we review key studies that have used EMA methods to investigate how dynamic changes in mood, energy levels, social safety, present focus, and burnout are associated with stress levels, well-being, and health.

### 3.1. Well-Being

Well-being is associated with health-promoting outcomes, including better physical and mental health [17]. Well-being contains both a subjective component, which is often assessed with life satisfaction and affect measures, and a psychological component, which is often conceptualized as thriving and finding meaning in life [18]. Rather than being just the absence of negative factors, well-being is better conceptualized as the presence of positive factors. Well-being predicts health, happiness, and longevity [17]. Although well-being is somewhat more stable than other psychological states (e.g., stress levels) over time, well-being also varies across time and contexts in ways that are overlooked by traditional single-timepoint well-being measures [19]. 

In studies specifically assessing within-person fluctuations in well-being, well-being is positively associated with being in nature and engaging in physical activity and negatively associated with being at work (for a review, see [14]). However, well-being in EMA studies is typically assessed with a brief measure of positive affect or subjective happiness [14,20], as opposed to a more comprehensive measure of eudemonic well-being that assesses aspects of growth, thriving, or meaning. Further, many studies have used limited statistical analyses that fail to isolate within-person effects, instead focusing on between-person effects or relations that collapse between- and within-person effects. This is unfortunate, as one of the greatest benefits of employing EMA methods is the rich, within-person data collected. Therefore, it is important to pair EMA data with complementary analytic methods to realize their full potential. 

### 3.2. Stress

Stress refers to a person’s subjective experience of being able to manage the tasks in their lives, and not to a specific stressful experience, which is called a stressor. Although a small to moderate amount of stress is generally considered to be normative, and even beneficial [21], high stress levels, especially over long periods of time, can negatively impact both physical and mental health [22,23]. Indeed, whereas acute stress is a very strong predictor of PTSD and depression, chronic stress dysregulates biological processes that cause or exacerbate a wide variety of conditions, including anxiety disorders, depression, psychosis, asthma, ulcers, diabetes, certain cancers, and autoimmune and neurodegenerative disorders [24,25]. In fact, nine of the top ten causes of death in the United States today are exacerbated or caused in part by stress [26].

Due to the high prevalence of chronic stress and its negative impact on health, many interventions have been designed to reduce stress levels and improve stress-related health outcomes (e.g., [27,28,29]). Assessing the success of these interventions is typically carried out using follow-up surveys administered 1–12 months following the completion of the intervention (e.g., [30]), in which participants indicate their stress levels over the last week or month. However, sparse assessments like these fail to capture the dynamic nature of stress, well-being, and health; moreover, they can be influenced by a person’s current psychological state in ways that can bias participants’ experiences over the last month. Infrequent assessments thus limit our understanding of how interventions impact people’s daily fluctuations in psychological states, preventing researchers from obtaining a mechanistic understanding of *how* interventions influence stress and well-being over time.

Comparatively less research has investigated daily fluctuations in stress levels (vs. well-being) in non-clinical populations, thus limiting our understanding of how stress varies across time and in response to changing circumstances. However, evidence is emerging that stress levels are especially dynamic [2,31,32], with a large amount of variability day by day, as well as over the course of a day. Further, stress levels are affected both by the number and severity of stressors experienced, along with a wide range of psychological states. Resilience, or how well people manage stressors, is associated with less negative affect in response to stressors (as assessed by EMAs), specifically in individuals who have experienced early life stress [33].

### 3.3. Mood and Affect

Positivity of mood and affect measures are so strongly related to well-being that they are often used as a proxy for assessing well-being itself. In college students, for example, researchers have found that daily levels of positive affect are negatively associated with both daily levels of self-reported stress and the perceived stress scale and positively associated with flourishing, a measure of well-being [34]. Beyond just levels of positive affect, variability in affect is also associated with well-being. Ong and Ram [1] reviewed research on how affective variability, instability, inertia, and reactivity influence health outcomes and well-being, above and beyond general levels of positive affect. They posited that those with fragile high positive affect (i.e., positive affect that, while sometimes high, is also highly variable) might not experience the same good health as those with more stable high positive affect, although this can be missed in traditional research designs. Consistent with this hypothesis, research using EMAs has found that decreases in the positivity of mood (i.e., positive affective reactivity) following a stressful life event predicted an increased mortality risk about ten years later, whereas neither negative affective reactivity to stressors nor general levels of positive affect influenced mortality risk [35]. Specifically, a one-unit increase in positive affective reactivity predicted a 132% increase in mortality risk. Studies such as these highlight the added value of using EMA methods to assess how daily fluctuations in mood and affect impact stress, well-being, and health. 

### 3.4. Social Safety

Humans are inherently social creatures, and our social interactions and their quality influence our stress levels and well-being by influencing perceptions of social safety [36,37,38]. Whereas social safety, connectedness, and inclusion predict positive life outcomes, loneliness, social isolation, and rejection predict negative health outcomes, reduced happiness, and reduced longevity [39,40,41,42]. Research using EMA methods and sensing data have revealed that daily social interaction (both conversation frequency and duration) are associated with lower levels of perceived stress, more so than levels of social interaction averaged across days [34], highlighting the advantages of assessing daily levels of social interaction using EMA methods. In a group of older adults living with HIV, researchers found that despite social interaction being associated with higher pain and fatigue ratings later that day, participants were happier when they spent time with others compared to when they spent time alone [43]. Likewise, by assessing participants three times per day for six days, researchers found that people felt more happiness and interest—and less sadness, tiredness, and pain—during assessments taken when they were engaged in a social interaction versus those taken when they were alone [44].

Finally, during COVID-19 lockdowns, one study [45] used EMA methods to investigate the impact of face-to-face social interactions on mood and stress in real time and found that having at least one social interaction preceding an assessment predicted a more positive mood, more calmness, heightened energetic arousal, and reduced stress compared to assessments when no social interactions were reported. These within-person effects persisted even while controlling for peoples’ moods and stress levels reported in the prior assessment, indicating that social interaction, and not pre-existing psychological states, were driving these effects. Because social interactions have a large impact on stress, well-being, and health, they are also important to consider in studies assessing health-related outcomes. Further, because social interactions vary in their frequency, duration, and quality across moments and days, EMA methods are especially well suited to assess social interaction and support in real time.

### 3.5. Energy and Arousal

Another state that varies considerably from hour to hour is energy and arousal levels. In children, researchers have found that high energy levels and low levels of tiredness over 30 min predict moderate to vigorous physical activity, which, in turn, predicts more positive affect, less negative affect, and increased energy [46]. One strength of using EMA methods to assess these associations is that researchers are able to understand the temporal relations between mental states and behaviors. In this study, for example, energy levels both predicted, and were predicted by, physical activity. Researchers have also used EMA methods to assess associations between exposure to childhood trauma and daily energy levels, finding that the more childhood trauma a person has experienced, the lower their momentary energy levels [47]. Additionally, those with more childhood trauma exposure also reported heightened anxiety, loneliness, perceived daily hassles, and use of maladaptive coping strategies, alongside reduced psychological well-being, life satisfaction, optimism, sense of coherence, self-efficacy, and perceived social support. One unexplored possibility is that daily energy levels may be one factor that mediates associations between childhood trauma exposure and mental health outcomes. 

As shown here, the use of EMA methods can help improve our understanding of complex associations between psychological states and stress, well-being, and health by revealing the exact temporal relations between these processes, and by facilitating the discovery of psychological mechanisms that might mediate or moderate associations between things like childhood trauma exposure and mental health outcomes. Further, EMA methods can help investigators determine how interventions impact people’s daily psychological states. For example, researchers [48] found that daily arousal was increased in participants who completed a meditation-based intervention after six months of training, postulating that elevated arousal might be one mechanism through which meditation training can improve people’s quality of life. EMA studies such as these can overcome temporal hurdles inherent to traditional studies which prevent the true assessment of mediation of effects, helping to uncover the mechanistic processes through which interventions or events influence later health outcomes. 

### 3.6. Present Focus and Mindfulness

Being focused on the present moment is generally associated with enhanced well-being [49]. Levels of present focus are positively associated with life satisfaction [50] and vary dynamically day by day in ways that predict daily fluctuations in well-being [51]. Mindfulness interventions are designed to increase present focus and have been found to be associated with reduced stress levels and greater acceptance and self-regulation [49], along with increased resilience, self-efficacy, and well-being [52,53]. Although the term mindfulness has become somewhat all-encompassing for many different types of psychological states and interventions, here, we define mindfulness as being focused on the present moment. Further, researchers have found that increased daily mindful states are related to enhanced coping and reduced appraisals of stress [54].

Other mental health interventions have also been found to increase present focus. For example, participants who engaged in three different types of meditation-based mental training interventions (presence-, affect-, and perspective-focused interventions) all exhibited elevated levels of present focus in their daily lives after three months of training, along with an increased ability to cope with everyday stressors [48]. Using EMA methods to assess present focus may be especially useful in intervention studies, insofar as present focus might mediate the association between the intervention and positive outcomes in daily life. 

### 3.7. Burnout

Burnout is a state of chronic physical and emotional exhaustion resulting from prolonged exposure to stressors that is often characterized by feelings of depersonalization and reduced personal accomplishment [55]. Burnout has become a significant concern in the workplace, with some studies reporting that more than half of American workers currently experience at least moderate levels of burnout [56]. Indeed, the demands of fast-paced work environments, long working hours, and increasing expectations have led to a rise in burnout rates among employees, negatively impacting their overall well-being and job performance [57], including reduced productivity, higher absenteeism, and increased turnover rates, leading to economic losses and compromised work culture [58].

Burnout is not only detrimental to psychological health [59], but also has profound implications for physical health. For example, persistent stress and emotional exhaustion associated with burnout have been related to weakened immune system function [60] and an increased risk of cardiovascular diseases [61]. Prior research in this context has used EMAs to measure burnout by asking participants to identify the extent of their emotional, physical, and mental exhaustion, along with end-of-day job satisfaction and quitting intentions [13]. By collecting real-time data through mobile devices, EMAs provide a dynamic way to track an individual’s stress levels, mood fluctuations, and daily work experiences. This research has helped to identify patterns of stressors and triggers that contribute to burnout, allowing for more targeted interventions and personalized support.

Beyond using EMAs to assess burnout, ecological momentary interventions (EMIs) may help prevent burnout before it occurs. EMIs, a term coined in 2005, are treatments provided to participants during their everyday life through a mobile device, either on their own or as a supplement to a different ongoing treatment [62]. EMIs intervene in one’s day and environment, encouraging a certain behavior or providing feedback in real time [63]. By delivering timely and context-specific support, EMIs provide employees with coping strategies, mindfulness exercises, and stress management techniques precisely when they are most needed. Such interventions can enhance self-awareness, foster resilience, and promote adaptive coping mechanisms [62] to reduce the risk of burnout and promote overall well-being in the workplace.

Because burnout is better conceptualized as the end of a continuum between rewarding and overwhelming work experiences rather than a discrete state [55], just-in-time interventions, or EMIs, might be especially useful in preventing negative outcomes associated with burnout. The integration of EMAs can aid in accurately measuring and understanding burnout dynamics, while EMIs offer a practical and proactive approach to prevent and address burnout effectively. Using these innovative technologies together could ultimately help promote more sustainable and supportive work environments, benefiting both employees and organizations alike.

## 4. Recommendations, Limitations, and Future Directions

As researchers have become increasingly aware that dynamic psychological states, such as acute stress and mood, and discrete experiences impact well-being and health, it is now clear just how important it is to assess these processes using methods that complement their natural dynamics. EMAs accomplish this by combining the ecological validity of real-time assessments in a participant’s natural environments with the flexibility to schedule data collection as frequently as is necessary to capture the temporal dynamics of variables of interest (e.g., several times per week to several times per day). This flexibility enables researchers to design well-powered studies with many participants simultaneously experiencing the same event (e.g., the transition to college, the start of an athletic season, a culturally significant holiday) that would otherwise be logistically impossible to conduct at scale using traditional lab visits. To aid readers in adopting the EMA approach in their own research, we conclude with some comments on general study recommendations and limitations. See Table 1 for examples of scales and stand-alone questions used in past EMA research. 

First, existing, non-EMA datasets can be leveraged to explore whether higher temporal resolution is necessary to reflect a process of interest (e.g., if the temporal stability or retest reliability is low, if standard data collection timescales do not reflect optimal time lags between variables of interest [85]). Second, researchers interested in EMA should explore different EMA-style designs to evaluate which are ideal for their specific research questions. Depending on the process of interest, it might be best assessed using a multiple-assessments-per-day EMA, a daily EMA, daily diary studies, or measurement burst designs, which combine EMAs with longer-term follow-up durations. Third, researchers should brainstorm other complementary methodologies to include in their EMA studies, such as wearable technologies, common stressor designs, geolocation, text-message mining, and multi-omics approaches [86].

Fourth, the optimal use of high-temporal-density data requires complementary statistical techniques. Although a full review of analytic options is outside the scope of this article (for a broad introduction to techniques tailored to EMA data, see [87]), we would like to particularly emphasize the potential of dynamic structural equation modeling due to its flexibility (see [88,89,90]). Additionally, data with at least 60 observations per individual are well suited for group iterative multiple model estimation (GIMME, [91], which provides information on both group-level and individual-level time series). Fifth, future stress researchers should consider combining “common stressor” designs—in which all participants experience the same stressful, naturally occurring event (e.g., job interviews, major life transitions, auditions)—with EMAs to evaluate effects of interest during ecologically valid (i.e., not laboratory-based) stressors. Finally, for the health-focused research reviewed above, results observed in non-clinical samples should be replicated in clinical samples and vice versa.

As the use of EMA methods continues to gain popularity, is important to emphasize several limitations of EMA data. First, frequent assessments increase participant burden, which must be considered during the study design (e.g., the length of surveys, survey prompt timing, compensation strategies). Second, the repetitive nature of the surveys can influence data quality; therefore, participant inattention checks (e.g., screening for items that would be rarely endorsed by the average participant) are critical for ensuring data quality [92]. Third, given the frequency of assessment, it is important to check participants’ compliance with an equally high degree of frequency—lest an extended lack of compliance result in a stretch of unusable data that diminishes the value of the rest of their data. Fourth, given the online nature of many EMA surveys, there are logistical challenges to conducting this research in areas of the world or in populations with limited internet access, which can present a challenge with both collecting EMA data using digital tools as well as with the generalizability of EMA-based research. Finally, although the benefits of real-time data collection are numerous, there are also some disadvantages to consider as well, depending on a researcher’s specific research question. For example, reports can be influenced by transient emotional responses or misunderstandings. Therefore, when feasible, collecting both EMA and retrospective data can be useful. 

## 5. Conclusions

In conclusion, EMA methods have the potential to reveal how dynamic fluctuations in psychological states impact stress, well-being, and health. In the present article, we examined their potential by reviewing studies that highlight how assessing aspects of mood, social safety, energy levels, present focus, and burnout using EMAs can reveal key associations that are missed in more traditional study designs. Assessing changes in these dynamics across the day, as well as across several days, can in turn help determine the temporal associations between health-relevant processes and elucidate psychological mechanisms through which interventions promote stress reduction and enhanced well-being.

In the future, using EMAs to assess factors that precede health problems will enable EMIs to be delivered in advance of those health issues developing, thus improving health and well-being. Along these same lines, EMAs that assess stress-related processes may be able to be harnessed as a therapeutic tool to aid individuals in enhancing resilience, enabling them to reflect on their reactions to stressors both in real time and using their own historical responses to strive to increase their own resilience. By providing examples of scales and single-item questions used in EMA studies, making concrete recommendations for researchers seeking to employ EMAs in their research designs, and discussing the limitations of EMA methods, we hope to encourage the use of these methods by researchers studying stress, well-being, and health. EMA methods are well poised to dramatically advance our understanding of the human experience and how such experiences impact human health and wellbeing. To fully realize this potential, EMA research will need to be carried out carefully, and in a manner that maximizes its scalability, generalizability, and acceptability to participants.

## Figures and Tables

**Table 1:** Examples of scales and stand-alone questions used for ecological momentary assessments (EMAs).

Construct	Scales	Stand-Alone Questions
Stress and Resilience	Perceived Stress Scale (PSS; [64]) Brief Resilience Scale (BRS; [33]) Connor–Davidson Resilience Scale (CD-RISC-10; [65])	*Rate your stress levels from 1 (not at all stressed) to 5 (extremely stressed)* [66] *How well are you coping with the challenges you’re currently facing?* [67] *Pick the image that best captures how stressed you feel right now*. [68]
Well-Being and Happiness	Satisfaction with Life Scale (SwLS; [69]) WHO-5 Well-being Index [67]	*All things considered, how satisfied are you with your life as a whole today?* [70] *How happy are you right now, on a scale from 0 (not at all) to 10 (completely)?* [71]
Mood and Affect	Positive and Negative Affect Schedule (PANAS; [72]) Self-Assessment Manikin (SAM; [73]) Profile of Mood States (POMS; [74])	*Rate your mood from 1 to 5 for the following: happy, angry, sad, stressed, worried*. [67] *Pick the image that best captures your mood right now*. [68] *How positive do you feel right now (cheerful, enthusiastic, awake, calm, relaxed)?* [75] *How negative do you feel right now (irritated, bored, nervous/stressed, distressed, depressed)?* [75]
Social Safety and Loneliness	Goldsmith Social Support Scale [76] UCLA 3-item Loneliness Scale [77]	*At the time of the prompt, were you having any social interaction?* [44] *Since the last alarm, how many times did you socialize with someone else (e.g., spent more than 5 min talking or communicating with someone else)?* [43]
Energy and Arousal	Multidimensional Fatigue Inventory (MFI; [78]) Subjective Vitality Scale [74]	*Rate your current energy level from 1 (no fatigue) to 10 (worst fatigue)* [79] *People often describe how they feel right now referring to the metaphor of a battery ranging from exhausted to full of energy. Please indicate which of the following battery icons describes your current state best. (pictorial scale;* [74])
Present-Focus and Mindfulness	Five Factor Mindfulness Questionnaire—Short Form (FFMQ-SF; [80]) Cognitive Affective Mindfulness Scale-Revised (CAMS-R; [81])	After recalling what they were thinking about when texted: *Which of the following would best characterize these thoughts? (past-focused, present-focused, future-focused)* [82]
Burnout	Maslach Burnout Inventory (MBI; [83]) Oldenburg Burnout Inventory (OLBI; [84])	*At this point in time, how emotionally exhausted are you from your day?* [13] *How accomplished do you feel in your work at this moment?* [13]

## Data Availability

Not applicable.
